# Quantitative selection of sample structures in small-angle scattering using Bayesian methods

**DOI:** 10.1107/S1600576724004138

**Published:** 2024-06-18

**Authors:** Yui Hayashi, Shun Katakami, Shigeo Kuwamoto, Kenji Nagata, Masaichiro Mizumaki, Masato Okada

**Affiliations:** a Graduate School of Frontier Sciences University of Tokyo Kashiwa Chiba277-8561 Japan; b Japan Synchrotron Radiation Research Institute Sayo Hyogo679-5198 Japan; c National Institute for Materials Science Tsukuba Ibaraki305-0047 Japan; d Facalty of Advanced Science and Technology Kumamoto University Kumamoto860-8555 Japan; Argonne National Laboratory, USA

**Keywords:** small-angle X-ray scattering, small-angle neutron scattering, nanostructure analysis, model selection, Bayesian inference

## Abstract

Small-angle scattering (SAS) is a key experimental technique for analyzing nanoscale structures in various materials. In SAS data analysis, selecting an appropriate mathematical model for the scattering intensity is critical, as it generates a hypothesis of the structure of the experimental sample. Traditional model selection methods either rely on qualitative approaches or are prone to overfitting. This paper introduces an analytical method that applies Bayesian model selection to SAS measurement data, enabling a quantitative evaluation of the validity of mathematical models. The performance of the method is assessed through numerical experiments using artificial data for multicomponent spherical materials, demonstrating that this proposed analysis approach yields highly accurate and interpretable results. The ability of the method to analyze a range of mixing ratios and particle size ratios for mixed components is also discussed, along with its precision in model evaluation by the degree of fitting. The proposed method effectively facilitates quantitative analysis of nanoscale sample structures in SAS, which has traditionally been challenging, and is expected to contribute significantly to advancements in a wide range of fields.

## Abstract 2

A Bayesian method is proposed for quantitatively selecting a mathematical model of a sample for small-angle scattering. The performance of this method is evaluated through numerical experiments on artificial data for a sample containing a mixture of multiple spherical particles.

## 1. Introduction

In recent years, the analysis of nanoscale structures of materials has become increasingly important in advancing the development of new materials and understanding biological phenomena. Small-angle scattering (SAS) is a fundamental experimental method for analyzing such nanoscale structures. It involves irradiating substances with X-rays or neutron beams and analyzing the resulting scattering intensity data at small angles, typically 5° or less (Guinier & Fournet, 1955 ▸). SAS is versatile and applicable to a wide array of heterogeneous materials including nanoparticles, polymers, soft materials and fibers, and is utilized across many fields including materials science, chemistry and biology.

SAS measurement data are expressed in terms of scattering intensity that corresponds to a scattering vector, a physical quantity representing the scattering angle. Data analysis requires selection and parameter estimation of a mathematical model of the scattering intensity that contains information about the structure of the specimen. This selection process is critical as it involves assumptions about the structure of the specimen.

Traditionally, model selection in SAS data analysis has been performed by listing candidate models according to theoretical or empirical rules, conducting parameter fitting against the measurements, and comparing suitability using criteria such as χ-squared error, among other criteria (Breßler *et al.*, 2015 ▸; Da Vela & Svergun, 2020 ▸; Kline, 2006 ▸; Larsen *et al.*, 2018*a* ▸; Pedersen, 1997 ▸; Schneidman-Duhovny *et al.*, 2010 ▸; Svergun *et al.*, 1995 ▸). Alternatively, models may be chosen on the basis of the general shape of the measurement data. However, these methods each have drawbacks: the former risks overfitting, which can lead to an overestimation of the model’s degrees of freedom (Rambo & Tainer, 2013 ▸), while the latter yields only qualitative model selections. Furthermore, quantitatively evaluating the reliability of the results is challenging with traditional methods.

In this study, we propose a novel framework for SAS model selection that quantitatively assesses the validity of mathematical models that represent specimen structures in measurements. This approach uses Bayesian model selection within the framework of Bayesian inference, a method increasingly applied to analysis of various types of physical experimental data (Nagata *et al.*, 2012 ▸, 2019 ▸; Rappel *et al.*, 2020 ▸; Machida *et al.*, 2021 ▸; Moriguchi *et al.*, 2022 ▸; Nagai *et al.*, 2021 ▸; Kashiwamura *et al.*, 2022 ▸; Katakami *et al.*, 2022 ▸; Nelson & Prescott, 2019 ▸; Orioli *et al.*, 2020 ▸; Scheres, 2012 ▸; Ueda *et al.*, 2023 ▸). In the context of SAS data analysis, Bayesian inference has been applied to various cases, including ensembles of protein structures (Antonov *et al.*, 2016 ▸), regularization methods in parameter fitting (Larsen *et al.*, 2018*b* ▸), indirect Fourier transforms (Hansen, 2000 ▸; Larsen & Pedersen, 2021 ▸), the estimation of particle size distributions (Asahara *et al.*, 2021 ▸) and specimen parameter estimates (Hayashi *et al.*, 2023 ▸). The method solves inverse problems by establishing the likelihood, which is the data generation model, and the prior distribution, which corresponds to the prior knowledge about the target being estimated. The posterior distribution is then calculated according to the model and parameters with the acquired data using Bayes’ theorem. In our proposed method, the posterior probability of the data generation model is calculated under the measured data using the exchange Monte Carlo method (Hukushima & Nemoto, 1996 ▸), also known as parallel tempering, and then the resulting values are compared among the candidate models while concurrently obtaining Bayesian estimates of the model parameters. Moreover, since the validity of the measured data model is obtained as a posterior probability, the reliability of the results can be quantified by comparing these probabilistic values.

We conducted numerical experiments to assess the effectiveness of our proposed method. These experiments are based on synthetic data used to estimate the number of distinct components in a specimen, which was modeled as a mixture of monodisperse spheres of varying radii, scattering length densities and volume fractions. The results demonstrate the high accuracy, interpretability and stability of our method, even in the presence of measurement noise. To discuss the utility of the proposed method, we compare our approach with traditional model selection methods based on the reduced χ-squared error.

The structure of this paper is as follows. We first formalize the proposed analytical method, and then describe the model of multicomponent monodisperse spheres used in our numerical experiments. In Section 4, we detail the setup and the results of these experiments using the proposed method to estimate the number of mixed components in the synthetic data. We then discuss the analytical capabilities of our method and the performance of the traditional method based on the degree of fitting. We conclude with implications and potential applications of our method.

## 2. Formulation of the proposed framework

In this section, we present a detailed formulation of our algorithm for selecting mathematical models for SAS specimens using Bayesian model selection. The pseudocode for this algorithm is provided in Algorithm 1.
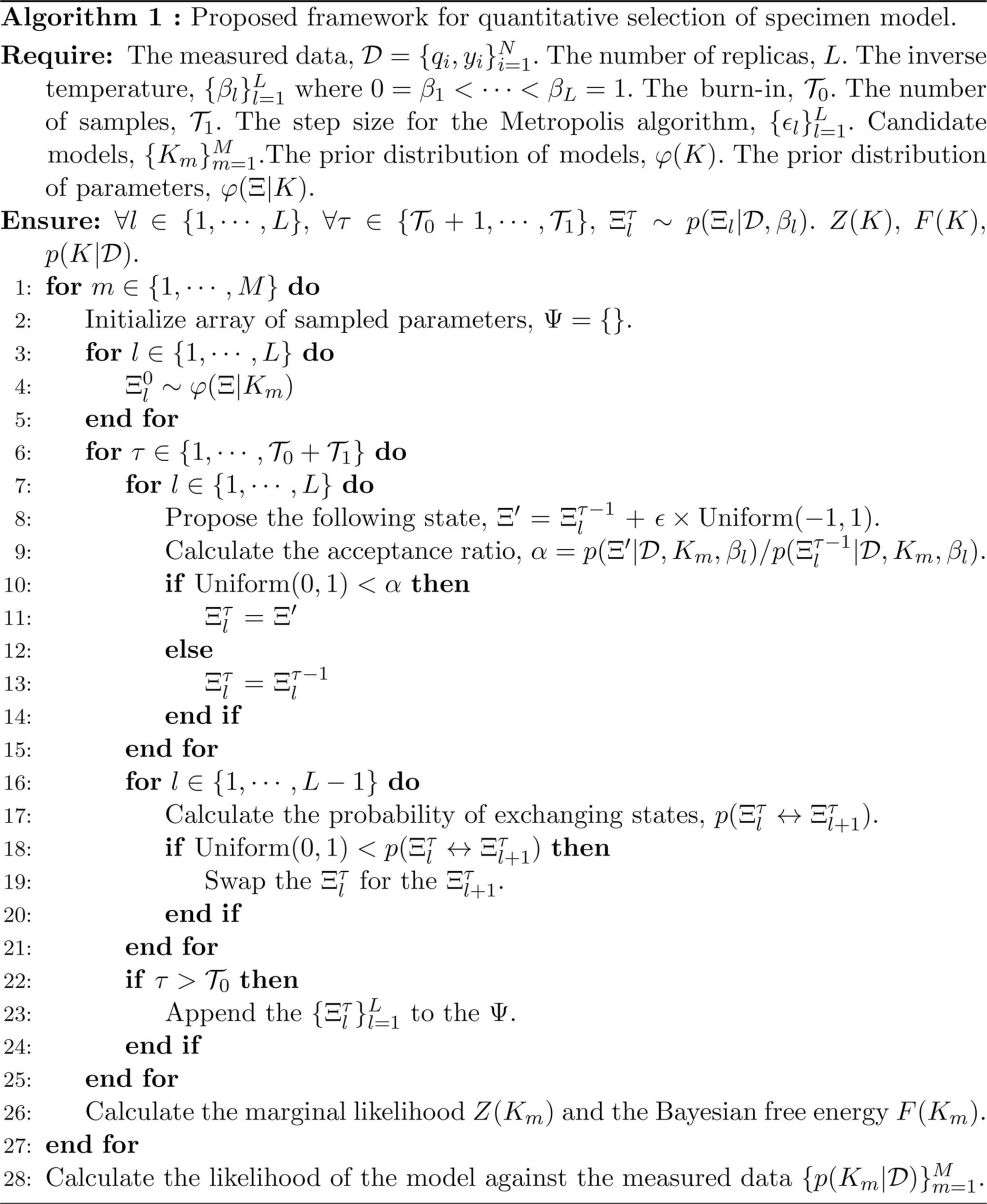


### 2.1. Bayesian model selection

The process of generating experimental measurement data is generally described by a probabilistic model that includes noise components. The SAS measurement data consist of scattering intensities that correspond to the scattering vector. As the scattering intensity is a measure of the number of incident photons on the detector, the scattering intensity values are assumed to follow a Poisson distribution (Katakami *et al.*, 2022 ▸; Kirian *et al.*, 2011 ▸; Liebi *et al.*, 2015 ▸; Nagata *et al.*, 2019 ▸). Let *I*_*K*_(*q*, Ξ) be the mathematical model of scattering intensity characterized by the parameter *K* for sample parameters Ξ and scattering vector magnitude *q*. The likelihood, which is the probability of generating the measured value *y*, is then given by
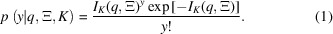
Assuming that the measurement data 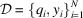, consisting of *N* data points, are samples from an independent and identically distributed population under *K* and Ξ, the likelihood is expressed by 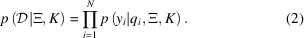
Here, we introduce the Poisson cost function to transform the likelihood of the measured data in equation (2) as
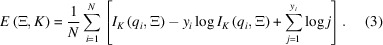
The likelihood is thus expressed as
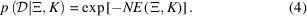


Let φ(*K*) be the prior distribution of the parameter *K* that characterizes the model, and φ(Ξ|*K*) be the prior distribution of the model parameters Ξ. Then, from Bayes’ theorem, the posterior distribution of the parameters given the measurement data can be written as
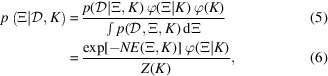

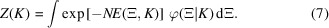
*Z*(*K*) is the marginal likelihood, which corresponds to the normalization constant of the posterior parameter distribution. The probability of model *K* given the data 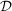, denoted 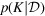, is given by
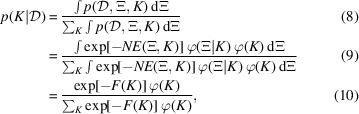
where
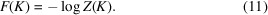
*F*(*K*) is referred to as the Bayesian free energy, also known as the stochastic complexity. The posterior probability of the model, 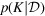, can be rephrased as the validity of model *K* for the measurement data 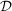. In other words, calculating and comparing the value of 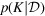 for all candidate models {*K*} thus enables quantitative model selection. Note that in Bayesian model selection the parameter *K* does not need to appear explicitly within the mathematical model of the specimen. This means that the method is also applicable to analyses such as comparing models with different sample shapes, like cylinders and ellipsoids, or evaluating the validity of spherical versus cylindrical models for the aspect ratios of colloidal particles.

### 2.2. Calculation of marginal likelihood

In our Bayesian model selection method, the Bayesian free energy *F*(*K*) and the probability 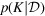 are calculated and compared for all candidate models. This computation relies on determining the marginal likelihood *Z*(*K*), as expressed in equation (7). The marginal likelihood generally involves multi-dimensional integration, which can be computationally intensive and unstable. To address this challenge, our framework uses replica-exchange Monte Carlo (REMC) to calculate the marginal likelihood (Hukushima & Nemoto, 1996 ▸). This method facilitates sampling from the desired probability distribution at multiple inverse temperatures, referred to as replicas, using the Markov-chain Monte Carlo method (MCMC) to exchange states strategically between adjacent inverse temperatures at arbitrary intervals, thus avoiding local minima. To calculate the marginal likelihood using REMC, we establish a series of *L* inverse temperatures 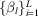 that satisfy the relation 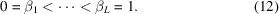
Sampling from the joint probability distribution at each inverse temperature gives
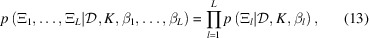
where Ξ_*l*_ denotes the model parameter at the *l*th inverse temperature β_*l*_. The posterior distribution 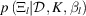 satisfies the following relation: 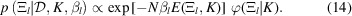
These distributions are sampled using MCMC at each inverse temperature, as expressed in equation (14), and states at adjacent inverse temperatures are periodically exchanged with a probability that satisfies the detailed balance condition. The probability of exchanging the *l*th and (*l* + 1)th states, *p*(Ξ_*l*_ ↔ Ξ_*l*+1_), is 
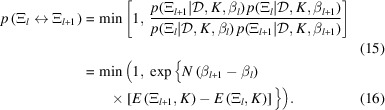


The marginal likelihood expressed in equation (7) can be efficiently determined using samples from various inverse temperatures sampled by REMC. The marginal likelihood *Z*(*K*, β) at inverse temperature β is expressed as 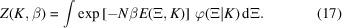
In this case, the target marginal likelihood expressed in equation (7) is equivalent to *Z*(*K*, β = 1). Using the relation in equation (12), *Z*(*K*, β = 1) can be expressed as follows: 
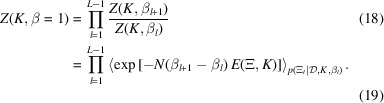
In equation (19), the symbol 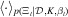 denotes the expectation value with respect to 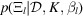. Computing equation (19) using sampling with REMC provides the marginal likelihood expressed in equation (7). Once the marginal likelihood *Z*(*K*) is determined, we can find the Bayesian free energy expressed in equation (11) and the posterior probability of model *K* given the measurement data expressed in equation (10). For the numerical experiments presented here, we used the Metropolis method (Metropolis *et al.*, 1953 ▸) for MCMC sampling of the posterior distributions at each inverse temperature, as expressed in equation (14).

### 2.3. Estimation of model parameters

During the marginal likelihood calculation the posterior distribution of 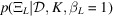 is obtained, which simply represents the Bayesian estimate of the model parameters (Hayashi *et al.*, 2023 ▸). Therefore, the parameter estimation is conducted simultaneously with performing the Bayesian model selection. Since the posterior distribution is sampled using REMC sampling, it can provide a global parameter estimate solution. The reliability of the estimation can be assessed from the statistical properties of the sampled posterior distribution.

In Bayesian estimation, the maximum *a posteriori* (MAP) solution provides a point estimate of the parameters. The MAP solution Ξ_MAP_ for the parameters of model *K* is expressed by this equation from equation (14): 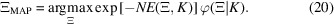


## 3. Formulation of a multicomponent monodisperse spheres model

In this section, we describe a model for the scattering intensity of a dilute sample comprising multicomponent monodisperse spheres (Guinier & Fournet, 1955 ▸; Hashimoto, 2022 ▸). This model serves as the basis for evaluating the performance of the proposed method.

Let 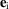 and 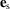 represent the unit vectors in the direction of the wavevector of the incident and scattered beams, respectively. If 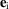 and 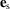 form an angle 2θ, and the wavelength of the beam is λ, then the scattering vector 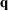 is given by
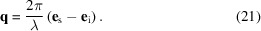
In this paper, we consider isotropic scattering and focus on the scattering vector’s magnitude *q*, defined as 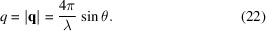


Monodisperse spheres are spherical particles of uniform radius. The scattering intensity *I*(*q*, ξ) of a specimen composed of sufficiently dilute monodisperse spheres of a single type for the scattering vector magnitude *q* is given by
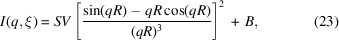
where 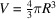. If the difference in scattering length density between the solute and solvent of the specimen is Δρ and the volume fraction is ϕ, then *S* = (3Δρ)^2^ϕ. The parameters ξ of this model are the particle size *R*, the scale *S* and the background *B*.

To formulate the scattering intensity of a specimen composed of *K* types of monodisperse sphere, we assume a dilute system and denote the particle size of the *k*th component in the sample as *R*_*k*_ and the scale as *S*_*k*_. The scattering intensity of a sample composed of *K* types of monodisperse sphere is then given by
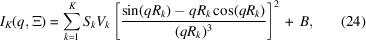
where we assume that 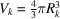. The model parameters Ξ for the scattering intensity *I*_*K*_(·) are 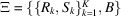.

## 4. Numerical experiments

**Figure 1:**
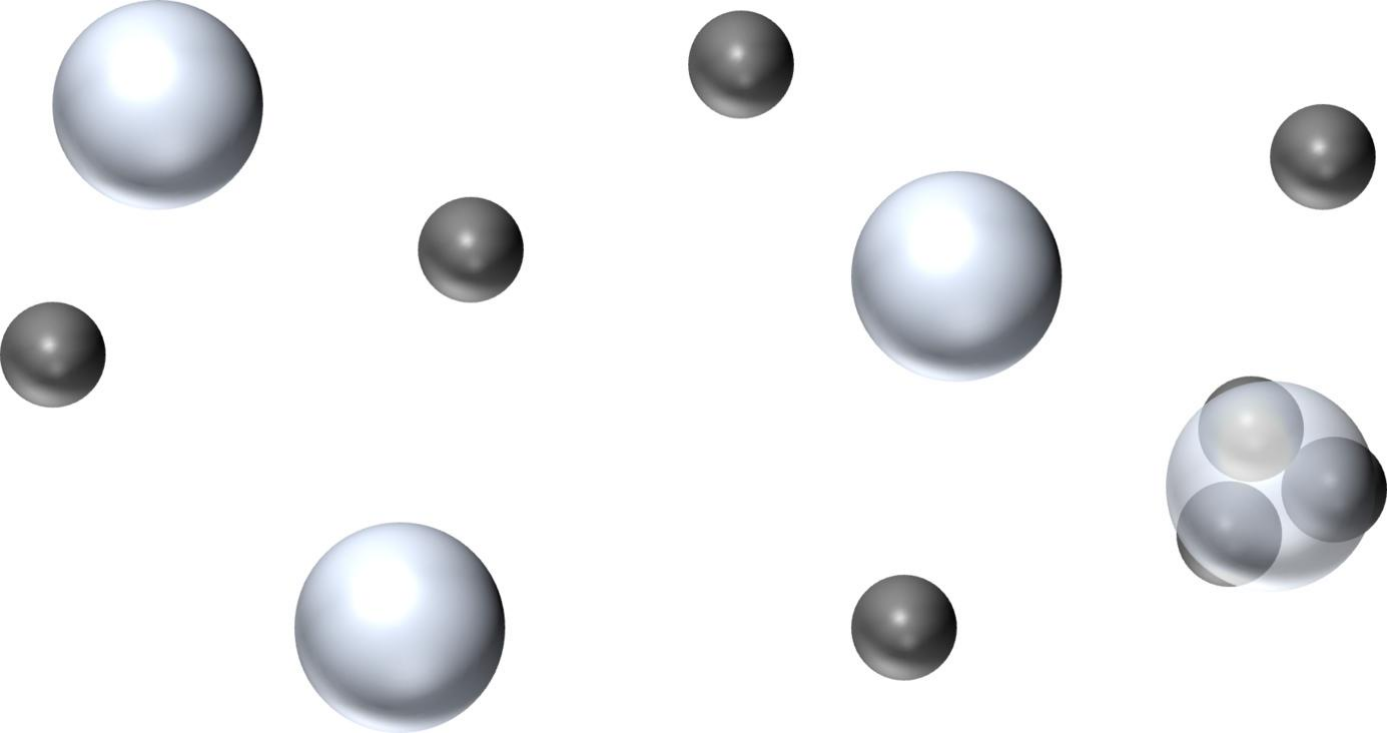
An illustration of a mixture of two types of spherical specimen. This shows scenarios with two components (*K* = 2), including mixtures of spherical particles of different sizes or volume fractions, and aggregates from a single particle type approximated as a large sphere.

Here, we present numerical experiments to evaluate the model selection among models with *K* ranging from one to four components to demonstrate the capabilities of the proposed framework. We apply the framework to synthetic data generated to represent a system with two types (*K* = 2) of monodisperse sphere, as described by equation (24). Bi­component spherical specimens, depicted in Fig. 1 ▸, correspond to model scenarios where two types of particle differing in size or volume fraction are mixed, or cases in which particles of a single type aggregate into larger spherical entities.

In typical SAS experiments, the scale parameter *S*_*k*_ in equation (24) tends to be small. Therefore, we normalize the scale parameter *S*_*k*_ as 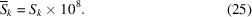
Accordingly, we refer to the model parameters as 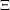 = 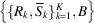.

The numerical experiments reported in this section were conducted with a burn-in period of 10^5^ and a sample size of 10^5^ for the REMC. We set the number of replicas for REMC, the values of inverse temperature and the step size of the Metropolis method taking into consideration the state exchange rate and the acceptance rate.

### 4.1. Generation of synthetic data

The scattering intensity in SAS experiments, which is typically recorded as count data, is subject to Poisson noise, as described by equation (1). We therefore generated synthetic data 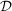 using the following procedure:

(i) Set the number of data points to *N* = 400 and define the scattering vector magnitudes at *N* equally spaced points within the interval [0.1, 3] to obtain 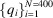 (nm^−1^).

(ii) Assume *K* = 2 and set the true model parameters Ξ to 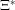.

(iii) Calculate the scattering intensity at the scattering vector magnitudes 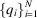 obtained in Step (i), using the model in equation (24) and 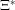. Introduce a pseudo-measurement time *T* to adjust the noise intensity in the data, to obtain 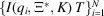.

(iv) Generate measurement values 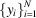 as Poisson-distributed random numbers with means of 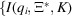
 
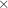

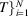
 to create the synthetic data set 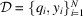.

In this section, we consider cases with pseudo-measurement times of *T* = 1 and *T* = 0.1. Generally, smaller values of *T* indicate greater effects from measurement noise.

### 4.2. Setting the prior distributions

In the Bayesian model selection framework, prior knowledge concerning the parameters Ξ and the model-characterizing parameter *K* is set as their prior distributions.

**Figure 2:**
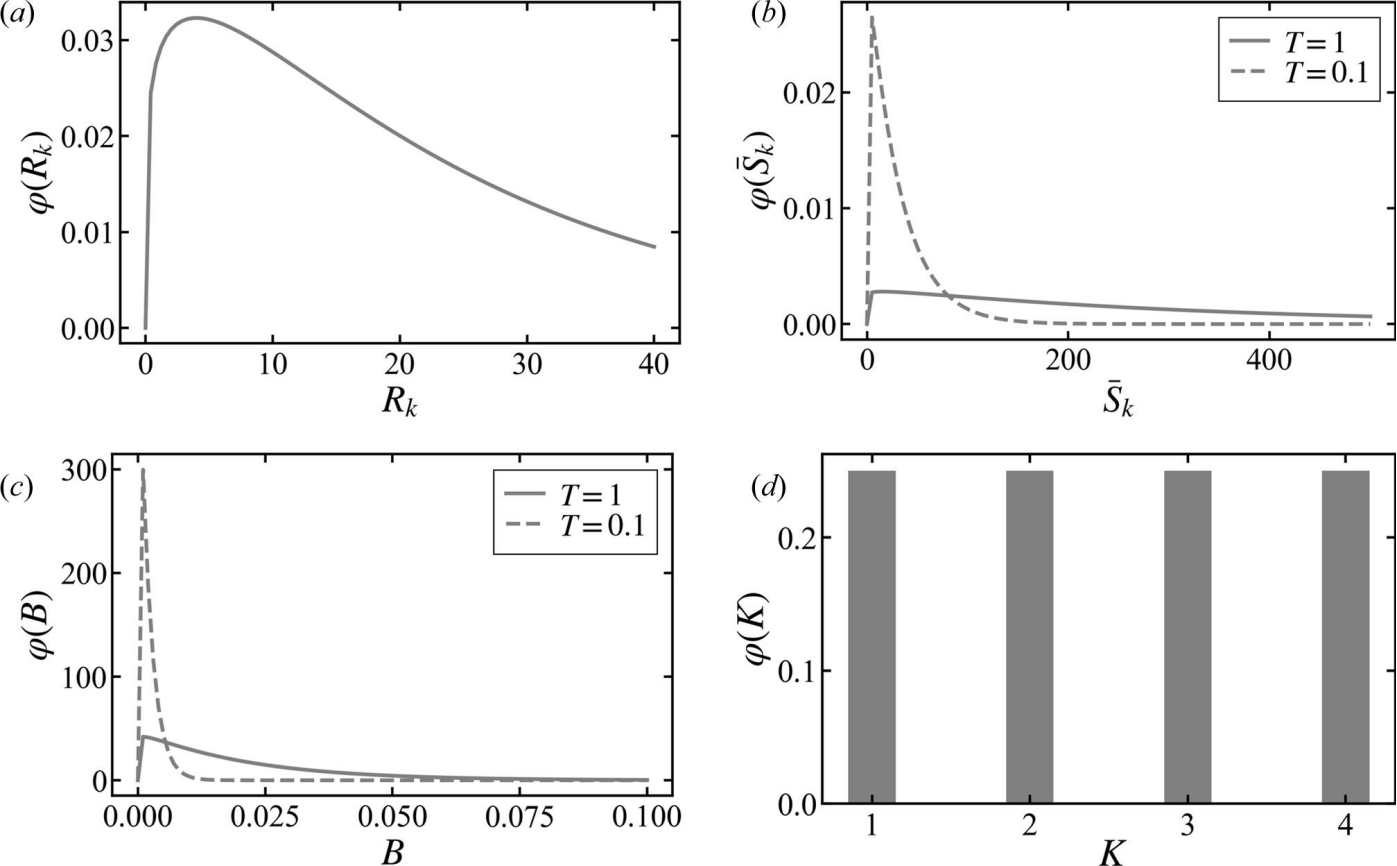
Plots of the prior distributions for various parameters. (*a*) Prior distribution of *R_k_*, φ(*R_k_*). (*b*) Prior distribution of 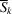, 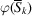. (*c*) Prior distribution of *B*, φ(*B*). (*d*) Prior distribution of *K*, φ(*K*).

In this numerical experiment, the prior distributions for the parameters Ξ were set as Gamma distributions based on the pseudo-measurement time *T* used during data generation, while the prior for *K* was a discrete uniform distribution over the interval [1, 4]. 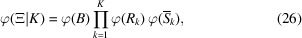

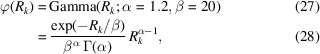

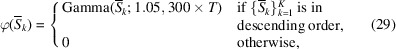

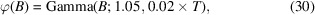

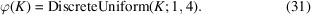
Fig. 2 ▸ shows the prior distributions for each parameter, as described in equations (28), (29), (30) and (31).

### 4.3. Results for two-component monodisperse spheres based on scale ratio

The ratio of the scale parameters *S*_1_ and *S*_2_ for spheres 1 and 2 during data generation, denoted *r*_*S*_, is defined as
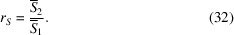
Next, we present the results from applying our proposed method to analyzing six types of data generated by varying the value of *r*_*S*_ for pseudo-measurement times of *T* = 1 and 0.1. Table 1 ▸ displays the parameter values used to generate the synthetic data.

**Figure 3:**
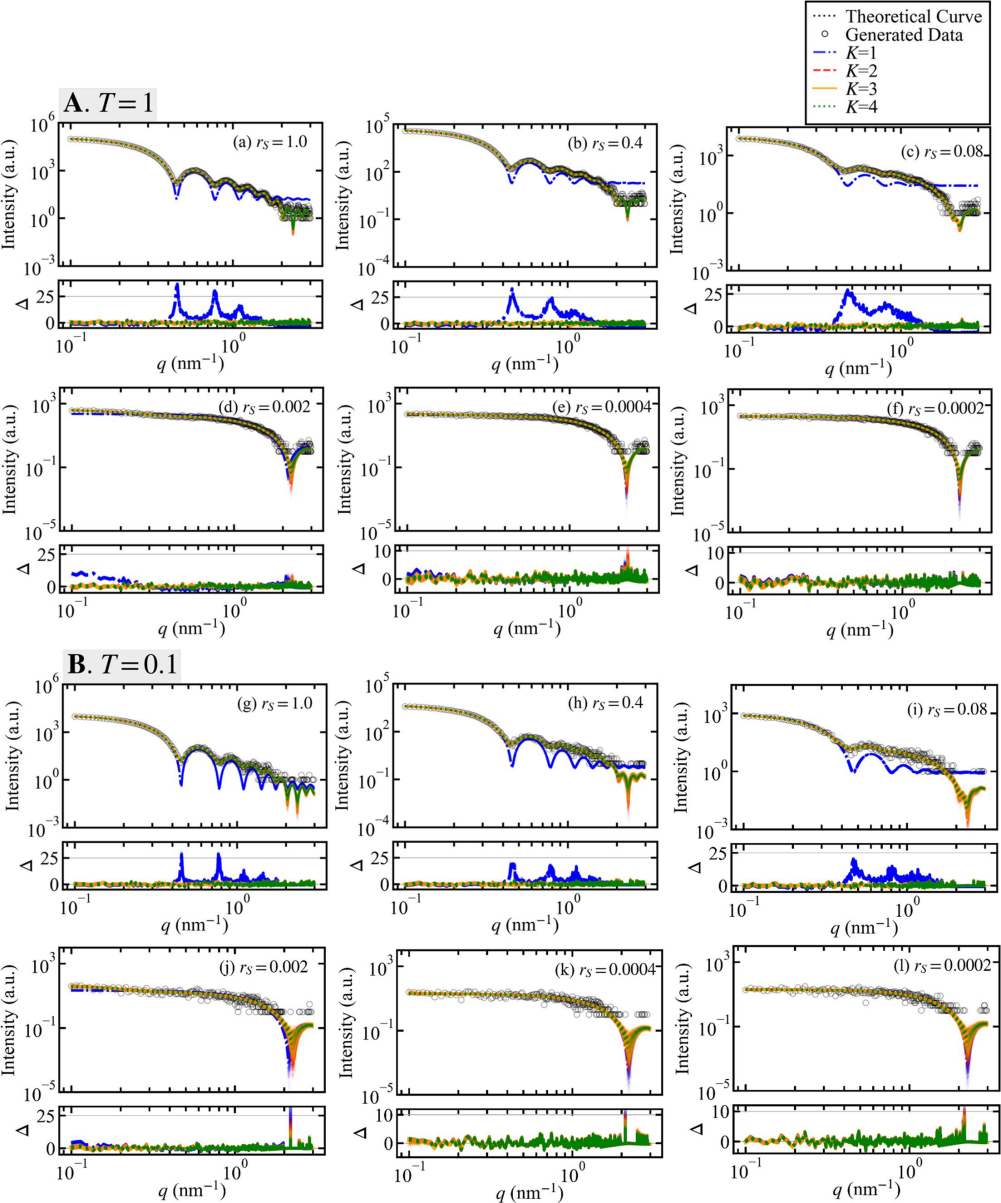
Fitting to synthetic data generated at various *r*_*S*_ values and residual plots. Panels **A** and **B** show cases for pseudo-measurement times of *T* = 1 and *T* = 0.1, respectively. In plots (*a*)–(*f*) and (*g*)–(*l*), the scale ratio *r*_*S*_ is displayed in descending order for *T* = 1 and *T* = 0.1, respectively. Black circles represent the generated data and the black dotted lines indicate the true scattering intensity curves. For models *K* = 1, *K* = 2, *K* = 3 and *K* = 4, the fitting curves and residual plots are represented by blue dashed–dotted lines, red dashed lines, orange solid lines and green dotted lines, respectively. Fitting curves were plotted using 1000 parameter samples that were randomly selected from the posterior probability distributions for each model. The width of the distribution of these fitting curves reflects the confidence level at each point.

Fig. 3 ▸ displays the fitting results and residual plots for synthetic data generated with the parameter values from Table 1 ▸. For each model (*K* = 1, *K* = 2, *K* = 3 and *K* = 4), 1000 samples were randomly selected from their respective posterior parameter distributions to plot these curves. Here, the residual Δ, which normalizes the difference between the model predictions and the observed data points (*q*, *y*) using the model parameters Ξ, is given by 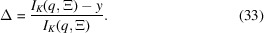
The fitting curves in Fig. 3 ▸ illustrate that the intensity and spread of these curves are indicative of confidence levels, where darker areas and narrower spreads denote higher confidence levels for the respective model.

In Fig. 3 ▸ the plots labeled (*a*) to (*c*) and (*g*) to (*i*) demonstrate, through the residual plots, that the model with *K* = 1 predominantly fails to represent the data accurately. However, we can also see that the fitting curves for models with *K* = 2–4 are almost identical in shape. The data shown in plots (*d*)–(*f*) and (*j*)–(*l*) are difficult to distinguish from the well known scattering data of a single type of monodisperse sphere (*K* = 1), making it challenging to qualitatively compare the goodness of fit among the models with *K* = 1–4.

**Figure 4:**
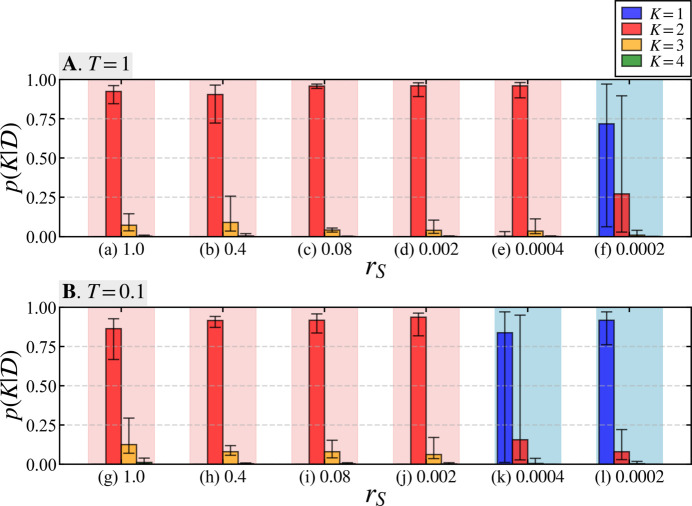
Results of Bayesian model selection among models *K* = 1–4 for varying *r*_*S*_ values. Panel **A** shows the posterior probability for each model using data generated with a pseudo-measurement time of *T* = 1, and panel **B** shows results for *T* = 0.1. In cases (*a*)–(*f*) and (*g*)–(*l*), the scale ratio *r*_*S*_ is displayed in descending order for *T* = 1 and *T* = 0.1, respectively. The height of each bar corresponds to the average values calculated for ten data sets generated with different random seeds, with maximum and minimum values shown as error bars. Areas highlighted in red indicate cases where, on average, the highest probability was found for the true model with *K* = 2, while blue backgrounds indicate that models other than *K* = 2 were associated with the highest probability on average.

Fig. 4 ▸ presents the Bayesian model selection results using our proposed framework. Within Fig. 4 ▸, panel **A** contains results for the case with *T* = 1 and panel **B** contains results with *T* = 0.1, each showing the probability 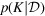 of model *K* based on the synthetic data 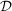 for each scale ratio *r*_*S*_. Here, ten data sets were created for each parameter value by varying the random seed during data generation, and the average value of 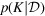 is indicated by the height of the bar graph, with error bars indicating the maximum and minimum values. For the relatively large scale ratios *r*_*S*_ in plots (*a*)–(*e*) in panel **A**, the true model with *K* = 2 has a high probability, while the average value of 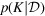 is highest for *K* = 1 in plot (*f*). In panel **B**, the true model with *K* = 2 is associated with high probability in cases (*g*)–(*j*), while in cases (*k*) and (*l*) *K* = 1 is associated with the highest probability.

Table 2 ▸ summarizes the number of times each model was found to have the highest probability in numerical experiments using the ten separate data sets shown in Fig. 4 ▸. For values of *r*_*S*_ = 0.0004 and above (Table 2 ▸, part **A**) and for *r*_*S*_ = 0.002 and above (Table 2 ▸, part **B**), the model with *K* = 2 was associated with the highest probability in all ten data sets. This demonstrates the high accuracy of the proposed method and its robustness to measurement noise. In cases (*f*), (*k*) and (*l*) of Fig. 4 ▸ and Table 2 ▸, the model with *K* = 1 was found to have the highest probability in nearly all of the ten data sets. These results were used to inform a discussion of the suitable analysis range of *r*_*S*_ using the proposed method, as addressed in the next section.

### 4.4. Results for two-component monodisperse spheres based on radius ratio

During synthetic data generation, the ratio of the radii *R*_1_ and *R*_2_ of spheres 1 and 2, denoted *r*_*R*_, was defined as
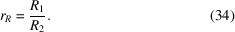
In this setup, we generated seven types of data by varying the value of *r*_*R*_ for pseudo-measurement times of *T* = 1 and *T* = 0.1.

**Figure 5:**
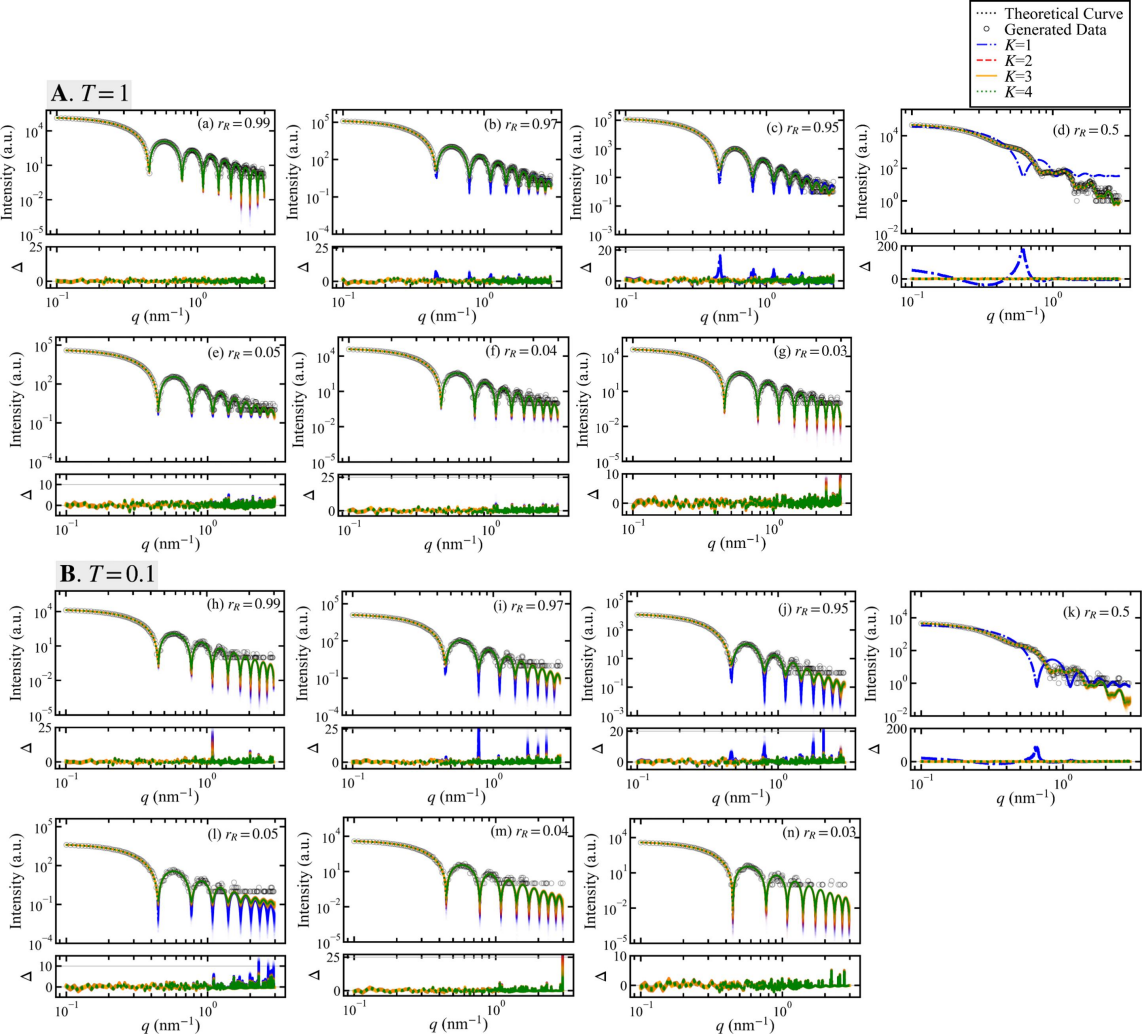
Fitting to synthetic data generated at various *r*_*R*_ values and residual plots. Panels **A** and **B** show cases for pseudo-measurement times of *T* = 1 and *T* = 0.1, respectively. In plots (*a*)–(*g*) and (*h*)–(*n*), the radius ratio *r*_*R*_ is displayed in descending order for *T* = 1 and *T* = 0.1, respectively. Black circles represent the generated data and the black dotted lines indicate the true scattering intensity curves. For models *K* = 1, *K* = 2, *K* = 3 and *K* = 4, the fitting curves and residual plots are represented by blue dashed–dotted lines, red dashed lines, orange solid lines and green dotted lines, respectively. Fitting curves were plotted using 1000 parameter samples that were randomly selected from the posterior probability distributions for each model. The width of the distribution of these fitting curves reflects the confidence level at each point.

Fig. 5 ▸ displays fitting curves and residual plots for the models *K* = 1, *K* = 2, *K* = 3 and *K* = 4, calculated from 1000 samples randomly selected from their respective posterior parameter distributions. These samples are derived from synthetic data generated with the parameter values given in Table 3 ▸. The residuals Δ were calculated using equation (33).

Aside from the cases of *r*_*R*_ = 0.5 in plots (*d*) and (*k*), the profiles of the data in Fig. 5 ▸ are very similar to those of a single monodisperse sphere, and the fitting curves for models *K* = 1 to *K* = 4 are nearly identical in shape. In contrast, the data for cases (*d*) and (*k*) with *r*_*R*_ = 0.5 have a complex profile, and the model with *K* = 1 represents the data poorly.

**Figure 6:**
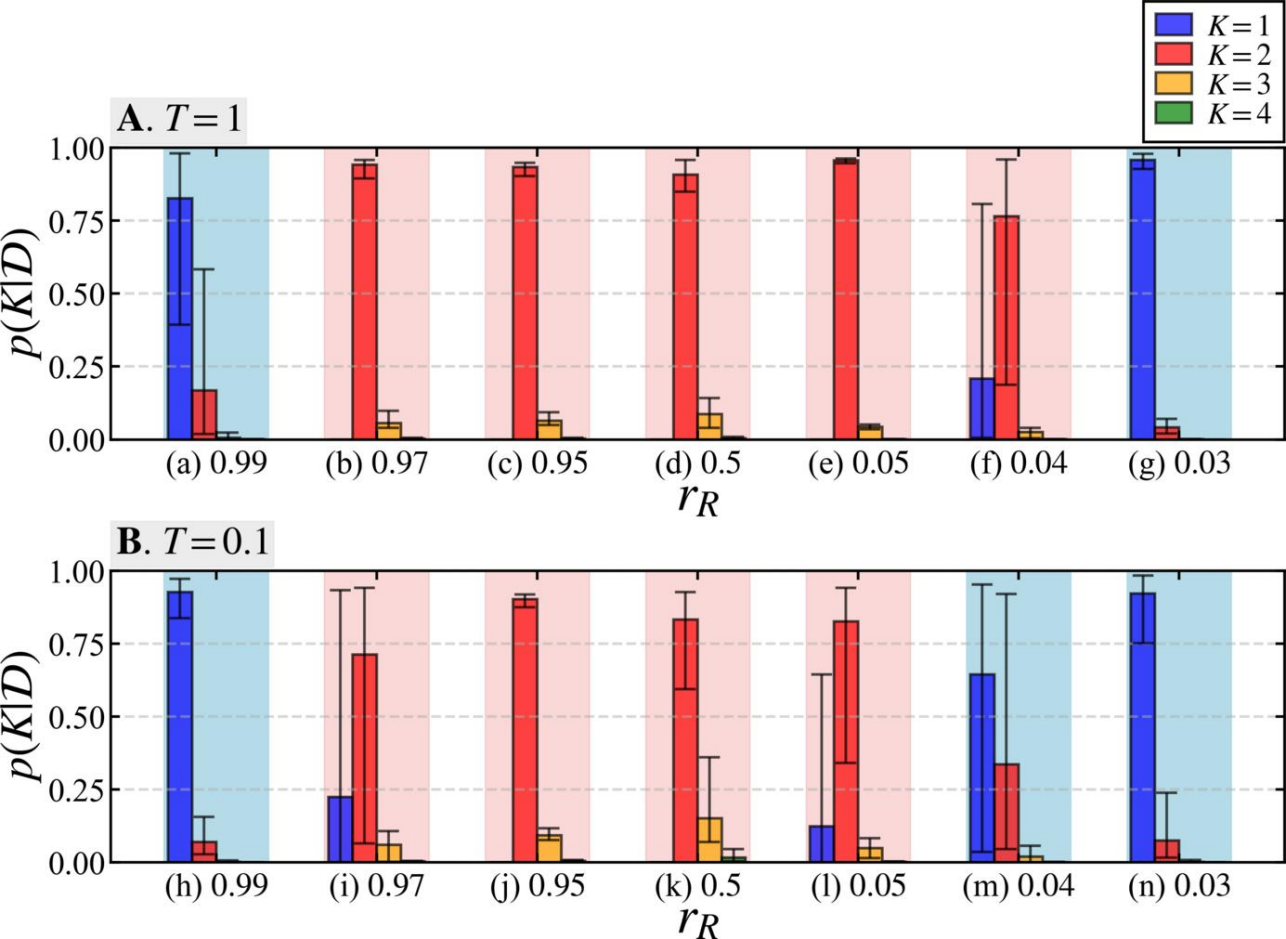
Results of Bayesian model selection among models *K* = 1–4 for varying *r*_*R*_ values. Panel **A** shows the posterior probability of each model using data generated with a pseudo-measurement time of *T* = 1, and panel **B** shows results for *T* = 0.1. In cases (*a*)–(*g*) and (*h*)–(*n*), the radius ratio *r*_*R*_ is displayed in descending order for *T* = 1 and *T* = 0.1, respectively. The height of each bar corresponds to the average values calculated for ten data sets generated with different random seeds, with the maximum and minimum values shown as error bars. Areas highlighted in red indicate cases where the true model *K* = 2 was most highly supported, while the blue backgrounds indicate that the likelihood of a model other than *K* = 2 was the highest.

Fig. 6 ▸ displays the results of Bayesian model selection using synthetic data generated by varying the radius ratio *r*_*R*_. Ten data sets were created for each parameter value by varying the random seed during data generation. The average value of 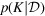 is indicated by the height of the bar graph, with the maximum and minimum values shown as error bars. Unlike the results for the variations in scale ratio shown in Fig. 4 ▸, the model selection procedure fails not only at a radius ratio *r*_*R*_ close to 0 but also at values close to 1, with *K* = 1 being the most highly supported. In the case of *r*_*R*_ = 0.04, the result for *T* = 1 in case (*f*) supports the true model *K* = 2, but for *T* = 0.1 in case (*m*) the alternative model *K* = 1 is the most highly supported. However, in cases (*b*)–(*f*) and (*i*)–(*l*), the true model *K* = 2 is associated with a high average probability (Fig. 6 ▸).

Table 4 ▸ presents the results of numerical experiments for the ten separate data sets shown in Fig. 6 ▸, summarizing the number of times each model *K* = 1–4 was most highly supported. Near the analytical limits of the proposed method, there are cases where the supported model changes depending on the data, as shown in Table 4 ▸, entries (*a*), (*f*), (*i*), (*l*) and (*m*).

## 5. Discussion

In Section 4, we conducted numerical experiments to determine the number of components *K* in two-component monodisperse sphere specimens using the proposed method through model selection applied to artificial measurement data. In this section, we discuss the analytical limits of our method under the settings of this study with respect to the scale ratio *r*_*S*_ and radius ratio *r*_*R*_ of the specimen’s two components, as well as the performance of the conventional model selection method based on the reduced χ-squared error.

### 5.1. Limitations of the proposed method

The experiments detailed in Section 4 explored the selection of the number of components *K* for two-component monodisperse spheres using the proposed Bayesian method. We observed certain analytical limitations for various values of the scale ratio *r*_*S*_ and radius ratio *r*_*R*_. In practical data analysis applications using the proposed method, it is advisable to conduct preliminary tests using synthetic data with noise intensity and anticipated parameter values similar to those of the measured data. This step can help ensure a more reliable analysis, as detailed below.

The scale parameter *S* is a value that is multiplied by the square of the difference in scattering length density between the solvent and the specimen, as well as the volume fraction. This can cause *r*_*S*_ to become extremely small when there is little difference in scattering length density between the solvent and a component of the specimen, or when there is a significant difference in the mixing ratio of the components. The results in Fig. 4 ▸ and Table 2 ▸ for a pseudo-measurement time of *T* = 1 (panel **A**) indicate that the model selection favored non-true models at a scale ratio of *r*_*S*_ = 0.0002. Similarly, for *T* = 0.1 (panel **B**), non-true models were favored at scale ratios of *r*_*S*_ = 0.0004 and *r*_*S*_ = 0.0002, indicating that these cases exceed the analytical capabilities of the proposed method. These findings imply that, within the experimental parameters of this study, the proposed method reliably identifies the true model with a high probability for scale ratios up to *r*_*S*_ = 0.0004 at *T* = 1 and up to *r*_*S*_ = 0.002 at *T* = 0.1.

In Section 4.4, we investigated the effect of varying the radius ratio *r*_*R*_. When components of different radii are mixed, it is important to consider not only simple mixtures but also instances of aggregated specimens. The findings shown in Fig. 6 ▸ and Table 4 ▸ indicate that the proposed method reaches its analytical limits as *r*_*R*_ approaches 1 and as it approaches 0. As *r*_*R*_ nears 1, the scattering profiles of the two-component system become similar to that of a single-component system, leading to the selection of the single-component model (*K* = 1). We found an analytical limit at *r*_*R*_ = 0.99 for both *T* = 1 and *T* = 0.1. The results for *r*_*R*_ = 0.97 show that at *T* = 0.1, which has a higher noise intensity than *T* = 1, the posterior probability of the single-component model (*K* = 1) increases, resulting in an unstable analysis. Conversely, as *r*_*R*_ approaches 0 with the results *r*_*R*_ = 0.03 at *T* = 1 and *r*_*R*_ = 0.04 and *r*_*R*_ = 0.03 at *T* = 0.1, the single-component model (*K* = 1) is associated with high probability, indicating an analytical limit. Overall, the proposed method demonstrates the ability to select the true model with high probability for radius ratios ranging from *r*_*R*_ = 0.04 to 0.99 at *T* = 1, and from *r*_*R*_ = 0.05 to 0.99 at *T* = 0.1.

### 5.2. Model selection based on χ-squared error

In SAS data analysis, selecting an appropriate mathematical model for the analysis is a crucial but challenging process. In this subsection, we compare the conventional model selection method based on the χ-squared error with the results of model selection using our proposed method.

Conventionally, model selection is performed by minimizing the χ-squared error through fitting with candidate models, and the model with a reduced χ-squared closest to 1 is considered to be the best representation of the data (Pedersen, 1997 ▸). The χ-squared error χ^2^ is given by the following equation: 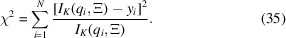
The reduced χ-squared 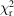 is obtained by dividing the χ-squared error by the degrees of freedom, dof: 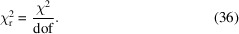
The degrees of freedom dof are calculated by subtracting the number of model parameters from the number of data points *N*. For the model represented by equation (24), it is given by
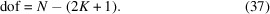


In the following, we discuss the results of selecting the model with the closest reduced χ-squared 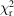 to 1 for models *K* = 1–4 using the same data generated with different random seeds for each of the six types of *r*_*S*_ determined by the parameters shown in Table 1 ▸ for *T* = 1, as in Section 4.3. Since it is difficult to obtain a global optimum solution using conventional fitting methods such as the quasi-Newton method and conjugate gradient method, we evaluate the reduced χ-squared 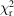 using the parameters that minimize χ^2^ among the parameters sampled from the posterior distribution in the experiment of Section 4.3.

**Figure 7:**
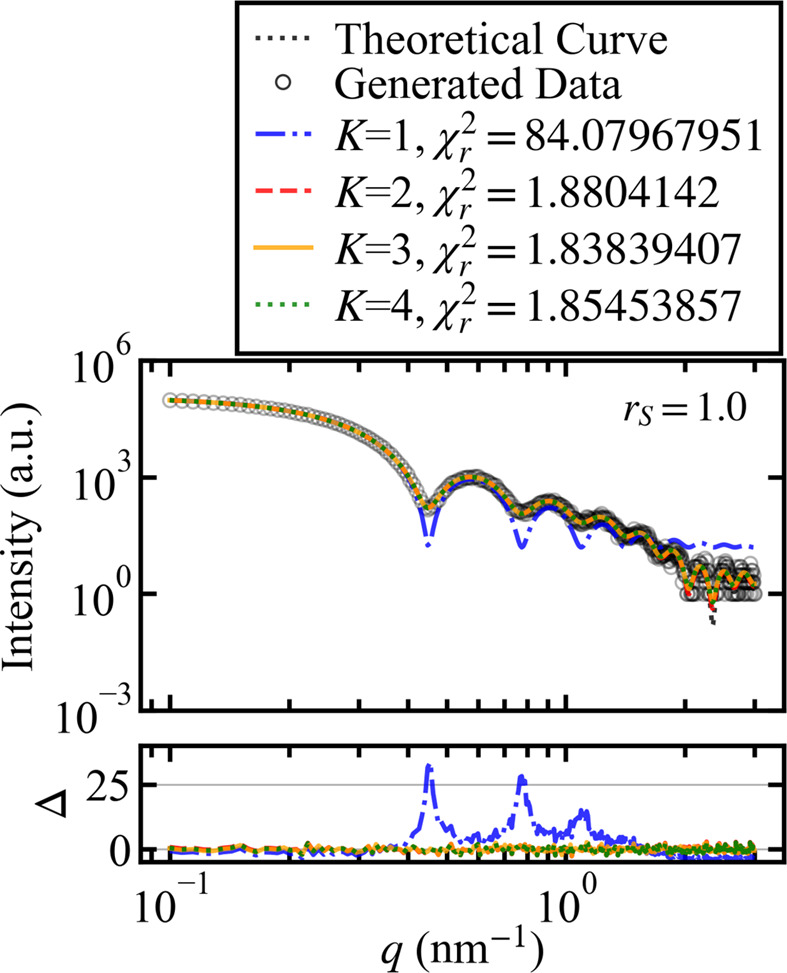
The fitting results and residual plots for the data shown in Fig. 3 ▸(*a*) were derived using parameters that minimize the χ-squared error from the posterior probability distributions for models ranging from *K* = 1 to *K* = 4. For each of these models, the fitting curves and their corresponding residual plots are represented by blue dashed–dotted lines, red dashed lines, orange solid lines and green dotted lines, respectively. The legend indicates the reduced χ-squared values for each model (*K* = 1 to *K* = 4).

First, we present the results for the data shown in Fig. 3 ▸ plot (*a*), generated using the model with *K* = 2. Fig. 7 ▸ shows the fitting results and reduced χ-squared for models *K* = 1–4 obtained by minimizing the χ-squared error for the data in Fig. 3 ▸ plot (*a*).

Fig. 7 ▸ shows the results obtained by the conventional method, indicating that the model with *K* = 3, even though it is not the true model, is considered most appropriate for the data due to its reduced χ-squared value being closest to 1. The fitting curves for models *K* = 2, 3 and 4 exhibit nearly identical shapes, which complicates the determination of the most suitable model based solely on their appearance.

Table 5 ▸ shows the aggregated results of calculating the reduced χ-squared for models *K* = 1–4 and counting the number of times each model was closest to 1 for the data sets (*a*)–(*f*) generated by varying the scale ratio *r*_*S*_ of the model with *K* = 2 in Section 4.3.

The results shown in Table 5 ▸ indicate that the model with *K* = 3 is the most supported for all data sets (*a*)–(*f*). This is thought to be because minimizing the χ-squared error failed to address the noise in the data adequately, ultimately leading to an overestimation of the model’s degrees of freedom. This implies that it is difficult to select the true model using the conventional method of comparing the 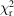 values among candidate models.

On the other hand, the results of the proposed method shown in Table 2 ▸ part **A** demonstrate that the true model *K* = 2 is supported ten out of ten times for cases (*a*)–(*e*). Within the analyzable range discussed in the previous subsection, the proposed method enables accurate model selection that takes into account the model degrees of freedom.

## 6. Conclusions

In this paper, we have introduced a Bayesian model selection framework for SAS data analysis that quantitatively evaluates model validity through posterior probabilities. We have conducted numerical experiments using synthetic data for a two-component system of monodisperse spheres to assess the performance of the proposed method.

We have identified the analytical limits of the proposed method, under the settings of this study, with respect to the scale and radius ratios of two-component spherical particles, and compared the performance of traditional model selection methods based on the reduced χ-squared.

The numerical experiments and subsequent discussion reveal the range of parameters that can be analyzed using the proposed method. Within that range, our method provides stable and highly accurate model selection, even for data with significant noise or in situations in which qualitative model determination is challenging. In comparison with the traditional method of selecting models based on fitting curves and data residuals, it was found that the proposed method offers greater accuracy and stability.

SAS is used to study specimens with a variety of structures other than spheres, including cylinders, core–shell structures, lamellae and more. The proposed method should be applied to other sample models to determine the feasibility of expanding the analysis beyond the case examined here to broader experimental settings. Future work could benefit from using the proposed method to conduct real data analysis, which is expected to yield new insights through our more efficient analysis approach.

## Figures and Tables

**Table 1:** Parameter values used for data generation with varying *r*_*S*_

	Sphere 1	Sphere 2
Radius *R* (nm)	2	10
Scale 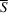	250	{250, 100, 20, 0.5, 0.1, 0.05}
Background *B* (cm^−1^)	0.01	
Pseudo-measurement time *T*	{1, 0.1}	

**Table:** (**A**) *T* = 1

	*K*			
*r*_*S*_	1	2	3	4
(*a*) 1.0	0	**10**	0	0
(*b*) 0.4	0	**10**	0	0
(*c*) 0.08	0	**10**	0	0
(*d*) 0.002	0	**10**	0	0
(*e*) 0.0004	0	**10**	0	0
(*f*) 0.0002	**8**	2	0	0

**Table:** (**B**) *T* = 0.1

	*K*			
*r*_*S*_	1	2	3	4
(*g*) 1.0	0	**10**	0	0
(*h*) 0.4	0	**10**	0	0
(*i*) 0.08	0	**10**	0	0
(*j*) 0.002	0	**10**	0	0
(*k*) 0.0004	**9**	1	0	0
(*l*) 0.0002	**10**	0	0	0

**Table 3:** Parameter values used for data generation when varying *r*_*R*_

	Sphere 1	Sphere 2
Radius *R* (nm)	{9.9, 9.7, 9.5, 0.5, 0.5, 0.4, 0.3}	10
Scale 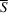	250	100
Background *B* (cm^−1^)	0.01	
Pseudo-measurement time *T*	{1, 0.1}	

**Table:** (**A**) *T* = 1

	*K*			
*r*_*R*_	1	2	3	4
(*a*) 0.99	**9**	1	0	0
(*b*) 0.97	0	**10**	0	0
(*c*) 0.95	0	**10**	0	0
(*d*) 0.5	0	**10**	0	0
(*e*) 0.05	0	**10**	0	0
(*f*) 0.04	1	**9**	0	0
(*g*) 0.03	**10**	0	0	0

**Table:** (**B**) *T* = 0.1

	*K*			
*r*_*R*_	1	2	3	4
(*h*) 0.99	**10**	0	0	0
(*i*) 0.97	2	**8**	0	0
(*j*) 0.95	0	**10**	0	0
(*k*) 0.5	0	**10**	0	0
(*l*) 0.05	1	**9**	0	0
(*m*) 0.04	**7**	3	0	0
(*n*) 0.03	**10**	0	0	0

**Table 5:** Model selection results based on reduced χ-squared values The table shows the number of times each model had the closest reduced χ-squared value to 1 for ten data sets generated with different random seeds for each *r*_*S*_ setting *T* = 1. Labels (*a*) to (*f*) refer to the settings in Figs. 3–4 and Table 2. The cases with the highest level of support for each data set are shown in bold.

	*K*			
*r*_*S*_	1	2	3	4
(*a*) 1.0	0	2	**8**	0\sim
(*b*) 0.4	0	0	**9**	1
(*c*) 0.08	0	0	**9**	1
(*d*) 0.002	0	0	**10**	0
(*e*) 0.0004	0	4	**5**	1
(*f*) 0.0002	0	2	**8**	0
